# Impact of Sports Medicine and Orthopedic Surgery Rotations on Musculoskeletal Knowledge in Residency

**DOI:** 10.7759/cureus.14211

**Published:** 2021-03-31

**Authors:** William Denq, James D Fox, Allison Lane, Beatrice Caballero, Brandon Godfrey, Jay Yim, Kate E Hughes, Thomas M Cahir, Anna Waterbrook

**Affiliations:** 1 Emergency Medicine/Sports Medicine, University of Arizona College of Medicine, Tucson, USA; 2 Family Medicine, University of Washington, Seattle, USA; 3 Emergency Medicine, University of Arizona College of Medicine, Tucson, USA; 4 Emergency Medicine, University of Arizona, Tucson, USA; 5 Health Sciences, University of Arizona, Tucson, USA

**Keywords:** sports medicine, orthopedic, residency, emergency medicine, musculoskeletal knowledge, assessment

## Abstract

Introduction

Musculoskeletal (MSK) complaints and injuries comprise 18.7% of emergency department visits. However, only 61% of emergency physicians (EP) pass a validated written Freedman and Bernstein MSK examination (FB-MSK). Educational interventions such as a primary care sports medicine (PCSM) rotation aid in MSK residency education. This study utilizes a validated MSK examination to evaluate and compare MSK knowledge acquisition following a traditional orthopedic rotation and a PCSM rotation.

Methods

Forty-nine interns were recruited to participate in this study over two academic years. The FB-MSK was administered to all participants at the start of residency. Participants were divided into two groups based on their residency sites; one group completed a traditional four-week orthopedic surgery rotation and the second group completed a four-week PCSM rotation. Forty-six of the forty-nine participants were administered the FB-MSK after completion of their rotations.

Results

Individual post-rotation scores significantly improved regardless of rotation (mean difference 2.78, *p<*0.001; 95% CI 2.05-3.52). The orthopedic surgery group significantly improved (mean difference 2.84, *p*<0.001; 95% CI 1.93-3.73) and the PCSM group significantly improved (mean difference 2.64, *p*=0.002; 95% CI 1.23-4.07). There was no significant difference in pre-rotation scores between the two groups (*p*=0.86; 95% CI -2.13 to 1.79). There was no significant difference in post-rotation scores between the two groups (*p*=0.66; 95% CI -1.98 to 1.26). There was no significant difference in mean score improvement between the two groups (*p*=0.81; 95% CI -1.33 to 1.69).

Conclusion

This study demonstrates significant MSK knowledge acquisition and no difference in the level of knowledge acquisition after completion of either traditional orthopedic surgery or PCSM residency rotation.

## Introduction

Musculoskeletal (MSK) complaints and injuries are a common reason patients seek medical care, comprising up to 18.7% of emergency department visits in the United States [1,2]. MSK conditions are prevalent in up to 33% of the general population. This in turn has a significant aggregate economic impact on the US healthcare system, costing $796 billion over a three-year period [3].

Despite the prevalence and high economic burden of these disorders, previous studies describe a deficiency in MSK education in the US medical system [4-11]. Medical students and residents report a lack of confidence in their mastery of MSK medicine [6-8]. A prior study reported 82% of medical school graduates failed a validated MSK examination [10]. Only 3% of preclinical curricula are spent teaching MSK pathology, highlighting the lack of training offered at multiple levels of medical education [9].

Among the variety of specialties that assess and manage MSK conditions, emergency physicians (EP) play a critical role. However, significant deficits exist in MSK knowledge at various career stages among EPs. As previously reported, only 61% of EPs passed a validated MSK examination [5]. The Model of Clinical Practice of Emergency Medicine, a foundation for undergraduate and graduate medical education, has two sections on traumatic and atraumatic MSK conditions commonly seen in the ED [12]. However, the Accreditation Council for Graduate Medical Education does not require nor mention MSK curricula for Emergency Medicine (EM) Residency Program accreditation [13]. A recent study demonstrated 56.1% of new EM residency graduates felt “not at all prepared” or “somewhat prepared” for MSK medicine [14]. The lack of a comprehensive MSK curriculum in EM training programs highlights the need for novel strategies to provide the essential and practical foundation of MSK education.

Waterbrook et al. describe substituting the standard orthopedic rotation with a primary care sports medicine (PCSM) rotation for EM residents [15]. The orthopedic rotation offered inpatient clinical time while the PCSM rotation offered outpatient clinical time and MSK examination and ultrasound education. This study reported higher resident satisfaction after the PCSM rotation. In addition, medical students and residents immersed in a week of interdisciplinary MSK education report increased confidence and demonstrate post-test MSK knowledge and MSK clinical skills competency [16]. An interdisciplinary and immersive MSK pilot program for continuing professional development may result in increased provider proficiency and confidence in MSK clinical skills [17].

There is very limited literature that assesses learners’ clinical knowledge before and after an MSK educational intervention. A validated MSK examination by Freedman and Bernstein (FB-MSK) exists to determine learners’ proficiency [10]. This study utilizes the FB-MSK to objectively assess participants’ clinical MSK knowledge. We aim to evaluate MSK knowledge of participating EM resident physicians before and after their PCSM or traditional orthopedic surgery rotation. We hypothesize that MSK knowledge acquisition, as defined by comparison of pre- and post-rotation examination scores, would not differ between the two rotation groups.

## Materials and methods

Forty-nine interns in two categorical Emergency Medicine Programs and one Combined Emergency Medicine/Pediatrics Program were recruited to participate in the study over two academic years from 2015 to 2017. The orthopedic surgery rotation occurred at a level one trauma center and the PCSM rotation occurred at a level four trauma center. The FB-MSK was administered at the beginning of both groups’ intern year and then again immediately after participants completed four-week intern rotations in either orthopedic surgery or PCSM. Participants were assigned rotations per their residency curriculum. The FB-MSK comprises 25 short-answer questions and a passing score, determined in the original study, is defined as 73.1% or above. The PCSM rotation included attendance at sports medicine clinics with primary care and orthopedic sports medicine physicians, involvement in sideline and event coverage, assigned reading materials, didactic experiences, and an on-call schedule to assist with reductions in the emergency department. The orthopedic surgery rotation was a traditional intern-level experience with the Department of Orthopedic Surgery’s inpatient and consultation service. The Institutional Review Board reviewed this project and was approved as exempt.

Data were entered into an Excel spreadsheet and summary statistics performed. Analysis was performed using the R program [18]. The FB-MSK pre- and post-test scores were compared using a paired t-test.

## Results

Forty-nine EM intern physicians participated in the study. The pre-rotation FB-MSK was completed by 49 residents. The post-rotation FB-MSK was completed by 46 residents. Two residents were lost to program attrition and one resident was unavailable in the orthopedic surgery group and was excluded from the analysis. The demographic data are summarized in Table 1 below.

**Table 1:** Demographic data (N=49) PCSM: primary care sports medicine; MSK: musculoskeletal; FB-MSK: Freedman and Bernstein validated examination

	Orthopedic rotation N=37 (%)	PCSM rotation N=12 (%)
Medical degree	37 (100)	12 (100)
Allopathic	36 (97.3)	12 (100)
Osteopathic	1 (2.77)	0 (0)
MSK rotation in medical school	14 (37.8)	2 (16.6)
Considering sports medicine as a subspecialty	5 (13.5)	1 (8.33)
Previous MSK certification	1 (2.70), certified personal trainer	0 (0)
Passed FB-MSK pre-rotation	9 (24.3)	4 (33.3)
Passed FB-MSK post-rotation	25 (67.6)	7 (58.3)

The mean pre-rotation score for the orthopedic surgery group was 65.8 ± 11.4% while the mean pre-rotation score for the PCSM group was 66.4 ± 11.7%. There was no statistically significant difference in pre-rotation scores between the two groups (*p*=0.86; 95% CI -2.13 to 1.79). The mean post-rotation score for the orthopedic surgery group was 77.8 ± 9.4% while the mean post-rotation score for the PCSM group was 76.3 ± 10.2%. There was no statistically significant difference in post-rotation scores between the two groups (*p*=0.66; 95% CI -1.98 to 1.26). There was no statistically significant difference in mean score improvement between the two groups (*p*=0.81; 95% CI -1.33 to 1.69). There was a statistically significant improvement in individual post-rotation scores regardless of rotation (mean difference 2.78, *p*<0.001; 95% CI 2.05-3.52). The orthopedic surgery group significantly improved (mean difference 2.84, *p*<0.001; 95% CI 1.93-3.73) and the PCSM group significantly improved (mean difference 2.64, *p*=0.002; 95% CI 1.23-4.07).

## Discussion

MSK knowledge is insufficient among many EPs and is frequently reported as an area for improvement by physicians [4-10]. To our knowledge, this is the first study to evaluate post-rotation MSK knowledge acquisition following a traditional orthopedic surgery or a PCSM rotation. Post-rotation scores improved from participating in either rotation with no statistically significant differences found. This demonstrates the effectiveness of both rotations at teaching MSK knowledge and also suggests that a PCSM rotation is a viable alternative to the traditional inpatient orthopedic surgery rotation. Although it is unclear why approximately one-third of interns failed post-rotation, it is clear that MSK knowledge transfer can be improved in both rotations.

Waterbrook et al. found increased resident satisfaction with this PCSM rotation compared to the orthopedic rotation [15]. The PCSM rotation emphasizes hands-on MSK clinical skills and provides one-on-one teaching with dually board-certified EM/Sports Medicine (SM) attending physicians. The EM/SM physician has the ability to educate EM resident physicians at the intersection of EM and MSK-related complaints. Training in a PCSM rotation that offers MSK ultrasound exposure may add an unmeasured benefit. Another potential benefit is the ease of access - scheduling within one department is often less complicated than with two. 

Although EM/SM educators may not be available at all institutions, a multi-specialty approach to developing an MSK curriculum for residents is feasible. Battistone et al. demonstrated that an MSK curriculum developed by multiple specialties led to a marked improvement in residents’ ability to evaluate and manage MSK complaints [16]. Gil et al. developed an orthopedic rotation in conjunction with EM and orthopedic departments and reported significant improvement in all 9 identified core content areas [19].

Consistent with previous data, the majority of interns at our institution failed the FB-MSK at the start of residency. Freedman and Bernstein reported 82% of new interns failed to demonstrate a passing level of orthopedic knowledge [10]. Subsequent studies reported 78% of internal medicine residents and 92% of fourth-year medical students failed the FB-MSK [20,21]. In concordance with these studies, our results add to previously published literature suggesting the underdevelopment of MSK education in undergraduate medical education. 

Day et al. developed an integrated preclinical MSK curriculum; post-implementation results revealed higher levels of clinical confidence in medical students [22]. DiGiovanni et al. found the only significant factor that leads to an increase in MSK knowledge was taking a two-week MSK- related elective [23]. Similar curricula should be considered in undergraduate medical education to ensure graduates have sufficient MSK knowledge and are increasingly confident as they enter residency.

Based upon data from this and our prior study evaluating both traditional inpatient orthopedic surgery and PCSM rotations for interns at our institution, we demonstrate both rotations significantly improving MSK knowledge, as measured by FB-MSK scores. Further studies should be performed to further assess MSK clinical skills and procedural competency following both types of rotations.

There are several limitations to this study. This is a single institution study with a small sample size that may limit its generalizability. While both rotations improved MSK knowledge, the results may simply reflect expected clinical advancement through residency. Given the sample size, there may be a significant difference between these rotations that could not be captured. The interns were evaluated immediately after their four-week rotation and anticipated progression through the intern year could not be measured. Additionally, there is the possibility that there was knowledge retention from the pre-test until the post-test as it was the same exam. This study does not evaluate retention beyond the intern year. The FB-MSK only assesses clinical knowledge and does not assess relevant physical examination or procedural skills. Although nationally validated and widely used, the FB-MSK was developed in 1998 [10]. Subsequently, Cummings et al. recently developed the MSK-30 in 2019; this is a validated exam aimed at assessing MSK knowledge in graduating medical students and primary care residents [24]. It is unclear if this tool may be more pertinent to EM residents compared to the orthopedic surgery-derived FB-MSK.

## Conclusions

This study demonstrates significant improvement in MSK knowledge after completion of both traditional orthopedic surgery and PCSM residency rotations. While no significant differences were found between the two rotations, it potentiates a PCSM rotation as a viable alternative to a traditional orthopedic rotation. EM Residency Programs should consider instituting a required PCSM or multispecialty developed MSK rotation to improve residents’ MSK knowledge and confidence.
